# Sex-related associations between body height and cognitive impairment among low-income elderly adults in rural China: a population-based cross-sectional study

**DOI:** 10.1186/s13293-021-00408-w

**Published:** 2021-12-06

**Authors:** Dongwang Qi, Chanhong Shi, Rongyan Mao, Xuewei Yang, Jinhui Song, Yanjia Wang, Jun Tu, Jinghua Wang, Xianjia Ning, Yi Wu

**Affiliations:** 1 Department of Neurology, Yiwu Central Hospital & The Affiliated Yiwu Hospital of Wenzhou Medical College, 699 Jiangdong Road, Yiwu City, 322000 Zhejiang China; 2 Department of Emergency Medicine, Yiwu Central Hospital &The Affiliated Yiwu Hospital of Wenzhou Medical College, 699 Jiangdong Road, Yiwu City, 322000 Zhejiang China; 3 Department of Neurology, Tianjin Medical University General Hospital, 154 Anshan Road, Heping District, Tianjin, 300052 China; 4 Laboratory of Epidemiology, Tianjin Neurological Institute, 154 Anshan Road, Heping District, Tianjin, 300052 China; 5 Tianjin Neurological Institute, Key Laboratory of Post-Neuroinjury Neuro-Repair and Regeneration in Central Nervous System, Ministry of Education and Tianjin City, 154 Anshan Road, Heping District, Tianjin, 300052 China

**Keywords:** Cognitive impairment, Body height, Sex difference, Epidemiology, Population-based study

## Abstract

**Background:**

Body height is a marker of childhood health and cumulative net nutrition during growth periods. However, sex-specific associations between body height and cognitive impairment are not well known in northern rural China.

**Methods:**

We assessed sex differences in the association between body height and cognitive impairment in a low-income elderly population in rural China. A population-based cross-sectional study was conducted from April 2014 to August 2014 to collect basic information from elderly residents aged 60 years and older in rural areas of Tianjin, China. Body height and Mini Mental State Examination (MMSE) scores were measured, and the relationships between these variables were assessed.

**Results:**

A total of 1081 residents with a mean age of 67.7 years were enrolled in this study. After adjusting for age, educational attainment, smoking status, drinking status, and the presence of hypertension, diabetes, and hypercholesterolemia, higher body height was found to be associated with a decreased prevalence of cognitive impairment in elderly men. Each 1-dm increase in height was associated with a 37% decrease in the prevalence of cognitive impairment. However, there was no significant association between body height and cognitive impairment among elderly women.

**Conclusion:**

In conclusion, shorter body height was related to cognitive impairment independently of age, educational attainment, lifestyle factors, and health-related comorbid factors among low-income elderly men in rural China. Accordingly, shorter elderly men may be targeted for effective dementia prevention in rural China.

## Background

Cognitive disorders, including dementia and mild cognitive impairment, are leading causes of disability and global public health priorities in aging populations [1, 2]. According to the World Alzheimer Report 2016, there were approximately 47 million patients with dementia worldwide, and this number is estimated to increase to more than 131 million by 2050. The total costs of dementia worldwide were approximately 604 billion US dollars in 2010 [3]. At present, most people with dementia live in developing countries, and this proportion is expected to increase to 71.2% by 2040 [4]. Moreover, the number of people living with dementia in China was approximately 10 to 12 million in 2014, which is the largest population worldwide and accounts for 25% of the total dementia population [5]. In China, the total costs of dementia were estimated to be 248.71 billion US dollars in 2020, posing a huge economic and social burden [5].

Although body height is affected by genetics and the environment, it is a measure of childhood health and cumulative net nutrition during growth periods [6]. Childhood height and leg length are predictors of intelligence and cognitive function in early life [7]. Most studies have reported positive relationships between body height and cognitive performance among elderly men in Western countries [8–12]. However, in Asia, only a few studies have been conducted in southern China and Korea, and the reported relationships between height and cognitive function are controversial. A cross-sectional study from the Guangzhou Biobank Cohort suggested that body height was positively associated with cognitive function, but only in older men and younger women [13]. Another community-based study indicated that shorter sitting height and longer leg length, rather than shorter body height, were associated with dementia among elderly women in urban Shanghai [14].

However, the relationships between body height in late life and cognitive impairment in low-income populations in northern rural China remain uncertain. In particular, sex-related associations between body height and cognitive impairment are not well known in northern rural China.

Therefore, the aim of this study was to evaluate the sex-related associations between body height and cognitive impairment among low-income elderly adults in northern rural China.

## Methods

### Study population

This was a population-based, cross-sectional study conducted from April 2014 to August 2014 in rural areas of Tianjin, China. The participants were from the Tianjin Brain Study, which has been described previously [15, 16]. All residents aged 60 years and older without vision and auditory dysfunction were invited to participate in this study. However, individuals with a history of myocardial infarction, stroke, congenital hypophrenia, and/or mental illness were excluded from the study.

### Risk factors and physical examinations

This study was conducted through face-to-face interviews by trained research staff. A pre-designed questionnaire was used to collect the following information: demographic information (including name, sex, date of birth, and education level), individual medical history (including the presence of hypertension and diabetes mellitus), and lifestyle factors (including smoking and drinking). A physical examination was performed to obtain information on body height, weight, and waist circumference (WC) with participants wearing thin clothes. Body height was measured in decimeters. Blood pressure (BP) measurements were performed using an electronic sphygmomanometer. Subjects were asked to remain resting in a sitting position for 15 min before testing; BP was measured three times and the mean was obtained. The levels of fasting blood glucose (FBG), total cholesterol (TC), triglycerides (TG), high-density lipoprotein cholesterol (HDL-C), and low-density lipoprotein cholesterol (LDL-C) were analyzed in the central laboratory of Tianjin Ji County People’s Hospital. Furthermore, cognitive function was measured using the Mini Mental State Examination (MMSE) owing to its high sensitivity and specificity in screening for cognitive impairment [17].

### Cognitive impairment criteria

The diagnostic criterion of cognitive impairment was based on MMSE score according to the participant’s education level. Cognitive impairment was defined by an MMSE score < 17 points in the group with no formal education (the illiterate group), < 22 points in the group with a primary school education, and < 26 points in the group with a junior school education and above [18].

### Definitions

Hypertension was defined by systolic blood pressure (SBP) ≥ 140 mmHg, diastolic blood pressure (DBP) ≥ 90 mmHg, the use of antihypertensive drugs, or a history of hypertension. Diabetes was defined as an FBG value ≥ 7.0 mmol/L, taking medication for diabetes, or a self-reported history of diabetes. Hypercholesterolemia was defined as a total cholesterol value > 5.72 mmol/L or taking lipid-lowering drugs. Smoking was defined as smoking ≥ 1 cigarette daily for more than 1 year. Drinking was defined as drinking > 50 mL of alcohol at least once per week for more than 6 months.

### Statistical analysis

Continuous variables are described as means and standard deviations; the Student’s *t* test was used to compare differences between two groups. Categorical variables are presented as numbers with frequencies, and the chi-squared test was performed to compare differences between two groups. The participants were categorized into three age groups (60–64, 65–69, and ≥ 70 years) and three education groups according to the length of formal education (0–5, 6–8, and ≥ 9 years). Multiple logistic regression analyses were used to evaluate the relationship between body height and cognitive impairment. The independent variables were selected as those found to be statistically significant in the univariate analyses. The relationship between body height and cognitive impairment was presented as adjusted odds ratios (ORs) and 95% confidence intervals (CIs) after adjusting for covariates. Three logistic regression analysis models were developed to examine the association between body height and cognitive impairment. Model 1 adjusted for age and educational attainment (categorical variables). Model 2 was based on model 1, with additional adjustment for lifestyle factors (smoking and drinking). Model 3 was based on model 2, with additional adjustments for hypertension, diabetes, and hypercholesterolemia.

All statistical analyses were performed with SPSS version 19.0 statistical software (SPSS Inc., Chicago, IL), and a two-sided *P* ≤ 0.05 was considered statistically significant.

## Results

### Demographic characteristics

A total of 1081 residents aged more than 60 years were enrolled in this study. There were 500 men (46.3%) and 581 women (53.7%), with mean age of 67.7 years overall, 68.0 years for men, and 67.5 years for women. In this rural population, the prevalence of cognitive impairment was 33.2% (26.6% in men and 38.9% in women). Moreover, the mean height was 15.9 dm (dm; 16.5 dm for men and 15.3 dm for women). In this population, women were more likely to have lower educational attainment and MMSE scores and higher DBP, FBG, TC, TG, HDL-C, and LDL-C levels (*P* < 0.05) (Table 1).

**Table 1:** Demographic characteristics and risk factors for all participants by sex group

Risk factors	Men	Women	Total	*P*
Total, *n* (%)	500 (46.3)	581 (53.7)	1081 (100)	–
Age, means (SD)	67.96 (6.42)	67.47 (6.48)	67.70 (6.46)	0.209
Age group, *n* (%)				0.209
60–64 years	190 (38.0)	249 (42.9)	439 (40.6)	
65–69 years	138 (27.6)	157 (27.0)	295 (27.3)	
≥ 70 years	172 (34.4)	175 (30.1)	348 (32.1)	
Education, years, mean (SD)	5.35 (2.72)	2.62 (2.94)	3.88 (3.15)	< 0.001
Education, *n* (%)				< 0.001
0–5 years	203 (40.6)	451 (77.6)	654 (60.5)	
6–8 years	219 (43.8)	101 (17.4)	320 (29.6)	
≥ 9 years	78 (15.6)	29 (5.0)	107 (9.9)	
Smoking, *n* (%)				< 0.001
No	136 (27.2)	261 (44.9)	397 (36.7)	
Yes	364 (72.8)	320 (55.1)	684 (63.3)	
Drinking, *n* (%)				< 0.001
No	280 (56.0)	492 (84.7)	772 (71.4)	
Yes	220 (44.0)	89 (15.3)	309 (28.6)	
Hypertension, *n* (%)				0.568
No	106 (21.2)	115 (19.8)	221 (20.4)	
Yes	394 (78.8)	466 (80.2)	860 (79.6)	
Diabetes, *n* (%)				0.012
No	432 (86.4)	469 (80.7)	901 (83.3)	
Yes	68 (13.6)	112 (19.3)	180 (16.7)	
MMSE score, mean (SD)	22.30 (4.54)	19.13 (5.31)	20.60 (5.21)	< 0.001
MMSE group, *n* (%)				< 0.001
Non-CI	367 (73.4)	355 (61.1)	722 (66.8)	
CI	133 (26.6)	226 (38.9)	359 (33.2)	
Body height (dm), mean (SD)	16.46 (0.56)	15.32 (0.55)	15.85 (0.80)	< 0.001
WC (cm), mean (SD)	88.58 (9.29)	89.21 (8.68)	88.92 (8.97)	0.250
SBP, mean (SD)	153.66 (23.06)	154.95 (23.28)	154.35 (23.18)	0.360
DBP, mean (SD)	87.05 (11.53)	85.52 (11.76)	86.23 (11.67)	0.031
FBG, mean (SD)	5.84 (1.18)	6.18 (1.93)	6.02 (1.64)	0.001
TC, mean (SD)	4.56 (0.95)	5.18 (1.07)	4.89 (1.06)	< 0.001
TG, mean (SD)	1.41 (0.83)	1.82 (1.29)	1.63 (1.12)	< 0.001
HDL, mean (SD)	1.42 (0.44)	1.53 (0.47)	1.48 (0.46)	< 0.001
LDL, mean (SD)	2.49 (0.79)	2.85 (0.90)	2.68 (0.87)	< 0.001

*SD* standard deviation; *MMSE* Mini-Mental state examination; *CI* cognitive impairment; *WC* waist circumstance; *SBP* systolic blood pressure; *DBP* diastolic blood pressure; *FBG* fasting blood glucose; *TC* total cholesterol; *TG* triglycerides; *HDL* high-density lipoprotein cholesterol; *LDL* low-density lipoprotein cholesterol

### Associations between cognitive impairment and risk factors by sex in the univariate analysis

Cognitive impairment was related to older age, lower educational attainment, and higher SBP in both men and women. Moreover, height and WC were associated with cognitive impairment in men (Table 2).

**Table 2:** Association of cognitive impairment with demographic characteristics and risk factors by sex in univariate analysis

Characteristics	Male			Female		
	Non-CI	CI	P	Non-CI	CI	P
Age, means (SD)	66.89 (5.71)	70.92 (7.31)	< 0.001	66.04 (5.36)	69.71 (7.41)	< 0.001
Age group, *n* (%)			< 0.001			< 0.001
60–64 years	158 (83.2)	32 (16.8)		175 (70.3)	74 (29.7)	
65–69 years	107 (77.5)	31 (22.5)		105 (66.9)	52 (33.1)	
≥ 70 years	102 (59.3)	70 (40.7)		75 (42.9)	100 (57.1)	
Education, years, mean (SD)	5.64 (2.57)	4.55 (2.97)	< 0.001	3.43 (2.99)	1.34 (2.36)	< 0.001
Education, *n* (%)			< 0.001			< 0.001
0–5 years	128 (63.1)	75 (36.9)		247 (54.8)	204 (45.2)	
6–8 years	180 (82.2)	39 (17.8)		84 (83.2)	17 (16.8)	
≥ 9 years	59 (75.6)	19 (24.4)		24 (82.8)	65 (17.2)	
Smoking, *n* (%)			0.121			0.672
No	93 (68.4)	274 (31.6)		157 (60.2)	104 (39.8)	
Yes	274 (75.3)	90 (24.7)		198 (61.9)	122 (38.1)	
Drinking, *n* (%)			0.922			0.536
No	206 (73.6)	74 (26.4)		298 (60.6)	194 (39.4)	
Yes	161 (73.2)	59 (26.8)		57 (64.0)	32 (36.0)	
Hypertension, *n* (%)			0.125			0.221
No	84 (79.2)	22 (20.8)		76 (66.1)	39 (33.9)	
Yes	283 (71.8)	111 (28.2)		279 (59.9)	187 (40.1)	
Diabetes, *n* (%)			0.081			0.325
No	323 (74.0)	109 (26.0)		282 (60.1)	187 (39.9)	
Yes	44 (74.8)	24 (25.2)		73 (65.2)	39 (34.8)	
Body height (dm), mean (SD)	16.51 (0.55)	16.31 (0.56)	< 0.001	15.35 (0.56)	15.29 (0.55)	0.191
WC (cm), mean (SD)	89.14 (9.26)	87.04 (9.25)	0.025	89.96 (9.00)	89.61 (8.15)	0.379
SBP, mean (SD)	152.27 (22.40)	157.49 (24.47)	0.025	152.95 (22.13)	158.10 (24.71)	0.009
DBP, mean (SD)	87.14 (10.91)	86.80 (13.12)	0.790	85.38 (11.41)	85.73 (12.31)	0.729
FBG, mean (SD)	5.79 (1.15)	6.01 (1.26)	0.064	6.23 (2.05)	6.11 (1.74)	0.467
TC, mean (SD)	4.55 (0.97)	4.57 (0.92)	0.812	5.15 (1.06)	5.20 (1.06)	0.400
TG, mean (SD)	1.42 (0.83)	1.41 (0.85)	0.790	1.86 (1.45)	1.75 (0.97)	0.346
HDL, mean (SD)	1.41 (0.42)	1.44 (0.49)	0.535	1.51 (0.44)	1.56 (0.50)	0.184
LDL, mean (SD)	2.49 (0.82)	2.48 (0.73)	0.883	2.84 (0.91)	2.87 (0.90)	0.699
MMSE score, mean (SD)	24.32 (2.69)	16.71 (3.87)	< 0.001	22.50 (3.20)	13.85 (3.27)	< 0.001

*SD* standard deviation; *MMSE* Mini-Mental state examination; *CI* cognitive impairment; *WC* waist circumstance; *SBP* systolic blood pressure; *DBP* diastolic blood pressure; *FBG* fasting blood glucose; *TC* total cholesterol; *TG* triglycerides; *HDL* high-density lipoprotein cholesterol; *LDL* low-density lipoprotein cholesterol

### Associations between height and cognitive impairment by sex and age in the multivariate analysis

Body height was associated with cognitive impairment among elderly men; each 1-dm increase in body height was associated with a 33% decrease (95% CI 0.45–0.99; *P* = 0.050) in the prevalence of cognitive impairment after adjusting for age, education group, WC, and SBP. In a subgroup analysis, the association remained consistent among adults aged 60–64 years. Each 1-dm increase in body height was associated with a 65% decrease (95% CI 0.17–0.74; *P* = 0.006) in the prevalence of cognitive impairment in elderly men aged 60–64 years.

Body height was related to the prevalence of cognitive impairment in models 1, 2, and 3 for elderly men. Each 1-dm increase in body height was associated with a 37% decrease (95% CI 0.43–0.94; *P* = 0.022) in the prevalence of cognitive decline after adjusting for age, education, smoking, drinking, hypertension, diabetes, and hypercholesterolemia in model 3. Each 1-dm increase in body height was associated with a 67% decrease (95% CI 0.16–0.69; *P* = 0.003) in the prevalence of cognitive decline for elderly men aged 60–64 years in model 3. However, there was no significant association between body height and cognitive impairment among elderly women (Table 3).

**Table 3:** Association of cognitive impairment with body height by sex and age, according to demographic characteristics and risk factor groups in the multivariate analysis

Body height	Model 1 OR (95% CI)	Model 2 OR (95% CI)	Model 3 OR (95% CI)
Male	0.61 (0.42, 0.90)*	0.61 (0.41, 0.90)*	0.63 (0.43, 0.94)*
60–64 years	0.34 (0.17, 0.69)*	0.34 (0.17, 0.71)*	0.33 (0.16, 0.69)*
65–69 years	1.14 (0.53,2.42)	1.12 (0.53, 2.39)	1.24 (0.57, 2.69)
≥ 70 years	0.69 (0.36, 1.30)	0.69 (0.36, 1.30)	0.74 (0.39, 1.43)
Female	1.07 (0.77, 1.49)	1.07 (0.77, 1.49)	1.08 (0.77, 1.50)
60–64 years	0.91 (0.52, 1.59)	0.91 (0.52, 1.59)	0.88 (0.50, 1.54)
65–69 years	0.87 (0.45, 1.70)	0.93 (0.47, 1.83)	0.93 (0.47, 1.85)
≥ 70 years	1.33 (0.78, 2.28)	1.28 (0.74, 2.20)	1.54 (0.86, 2.75)

Model 1 analysis adjusted by age group and education group;

Model 2 analysis adjusted by age group, education group, smoking and drinking;

Model 3 analysis adjusted age, education, smoking, drinking, hypertension, diabetes and hypercholesterolemia

*P ≤ 0.05

## Discussion

In the present population-based study, we evaluated the sex-related associations between body height and cognitive impairment among low-income elderly adults in rural China. We found that shorter body height was related to cognitive impairment independently of age, educational attainment, lifestyle factors, and health-related comorbid factors for elderly men, but not for elderly women. In a subgroup analysis, the association remained consistent among elderly men aged 60–64 years; each 1-dm increase in body height was associated with a 65% decrease in the prevalence of cognitive impairment.

Cardiovascular risk factors, including hypertension, diabetes mellitus, hyperlipidemia, and smoking, have been linked with cognitive decline in adults Childhood is a critical period for both brain and body development. Anthropometric measures, including head circumference and body height, are key indicators for brain and body development. A smaller head circumference was reported to be associated with poorer global cognitive performance and cognitive reserve [19, 20]. The Honolulu–Asia Aging Study suggested that height in middle life was a marker of childhood growth and was associated with dementia and cognitive impairment among elderly Japanese–American men living in Hawaii [7]. In a large Belarusian prospective birth cohort study, a taller height between birth and age 6.5 years was associated with higher cognitive scores at age 16 years, with a 2.5-point (95% CI 1.9, 3.0) per standard deviation increase [21]. Most studies in Western countries have suggested that height is associated with cognitive function in older age. A European study involving 11 countries reported that height was positively and significantly correlated with cognitive performance in later life, with each 10-cm increase in body height associated with a 0.04-standard deviation increase in a global cognitive score [8]. A cross-sectional study in a Portuguese community found that height was a good independent predictor of general cognitive function in later life, especially with respect to executive function and memory [9].

However, the relationships between body height in later life and cognitive impairment remain controversial in Asian countries. The Hallym Aging Study in Korea found sex-related associations between height and cognitive function. Among community-dwelling elderly men, compared with the tallest group, the shortest group had a 3.2-fold higher risk of cognitive impairment (OR 4.20; 95% CI 1.02–17.36), but this association was not present among elderly women [22]. A cross-sectional study from the Guangzhou Biobank Cohort suggested that body height was positively associated with cognitive function, but only in older men and younger women [13]. In addition, shorter sitting height and lower relative sitting height were found to be significantly associated with dementia among elderly women in the urban Shanghai community in China [14]. In the present study, body height had a positive association with cognitive function among low-income elderly men in rural China; however, no relationship was found for elderly women. Furthermore, another longitudinal Chinese study found that arm length was associated with cognitive impairment in men during a 3-year follow-up, but not in women [23].

Adult body height is the result of a combination of genetic and environmental factors [6]. Childhood environment, including nutrition and disease, accounts for 20% of variation in body height. Thus, body height is a good indicator of childhood living conditions. Early deficiencies in nutrition in childhood have adverse effects on physical growth and brain development, which in turn affect cognitive development [24]. Our findings suggest that shorter height and early-life nutritional deprivation may contribute to cognitive impairment among elderly men. A structural magnetic resonance imaging analysis performed in 515 middle-aged male twins revealed the mechanisms underlying this relationship: height was positively associated with total cortical surface area among male twins, which underlies the phenotypic height and general cognitive ability relationship [25]. Besides, male fetuses are more sensitive to disadvantageous prenatal conditions compared with girls in previous studies, which lead to lower birth weights and early life illness among men [26, 27]. Therefore, height might be a better indicator of early life conditions for men than women. Thus, there is still a need to elucidate the sex difference between body height and cognitive impairment.

Cardiovascular variables, including hypertension, diabetes, hyperlipidemia, and smoking, are risk factors for cognitive decline, while education is an independent protective factor against cognitive impairment and dementia in later life [28–30]. In a population-based study in a rural region of South Africa, short height in old age was associated with poor cognitive decline among those without a formal education but not among those with at least 8 years of formal education [31]. However, in the present study, we failed to find mediating effects of education on the height and general cognitive ability relationship. Consistent with our findings, height was significantly associated with cognitive function independent of education and other covariates in an English longitudinal survey of aging [32].

There are several limitations in this study. First, the study population was from a low-income, low-education, rural population in northern China, so its representation and generalizability are limited. Second, cognitive impairment was evaluated with MMSE scores; therefore, different types of cognitive impairment could not be further diagnosed in the study. Third, other confounding factors, including APOE4 genotype and diet, are important factors for cognitive decline, but they were not assessed in this study. Forth, low birth weight could be an important factor on cognitive and socioeconomic outcomes [33]. As all participants did not experience malnutrition at time of maternal pregnancy or the first years of life, birth weight or history of drought or famine was not included in present study. Finally, this was a cross-sectional study, so causal relationships between cognitive function and height could not be determined.

## Conclusion

In conclusion, shorter body height was associated with cognitive impairment independently of age, education, lifestyle factors, and health-related comorbid factors among low-income elderly men in rural China. The results suggest that childhood nutritional deprivation may contribute to cognitive impairment in later life. Accordingly, shorter elderly men may be targeted for effective dementia prevention in rural China. However, longitudinal cohort studies are warranted to confirm causality.

### Perspectives and significance section

It is crucial to focus on the status of nutrition among adolescent males for preventing against the cognitive impairment at adult stage, especially among low-income population.

## Data Availability

Please contact author for data requests.

## Abbreviations

MMSE: Mini Mental State Examination
WC: Waist circumference
BP: Blood pressure
FBG: Fasting blood glucose
TC: Total cholesterol
TG: Triglycerides
HDL-C: High-density lipoprotein cholesterol
LDL-C: Low-density lipoprotein cholesterol
SBP: Systolic blood pressure
DBP: Diastolic blood pressure
ORs: Odds ratios
CIs: Confidence intervals
